# Biosynthesis of Random-Homo Block Copolymer Poly[Glycolate-*ran*-3-Hydroxybutyrate (3HB)]-*b*-Poly(3HB) Using Sequence-Regulating Chimeric Polyhydroxyalkanoate Synthase in *Escherichia coli*

**DOI:** 10.3389/fbioe.2020.612991

**Published:** 2020-12-08

**Authors:** Shuzo Arai, Sayaka Sakakibara, Robin Mareschal, Toshihiko Ooi, Manfred Zinn, Ken’ichiro Matsumoto

**Affiliations:** ^1^Graduate School of Chemical Sciences and Engineering, Hokkaido University, Sapporo, Japan; ^2^Department of Engineering, Hokkaido University, Sapporo, Japan; ^3^Institute of Life Technologies, University of Applied Sciences and Arts Western Switzerland (HES-SO Valais-Wallis), Sion, Switzerland; ^4^Division of Applied Chemistry, Faculty of Engineering, Hokkaido University, Sapporo, Japan

**Keywords:** sequence regulation, block copolymer, engineered PHA synthase, polyglycolic acid, chimeric enzyme

## Abstract

Glycolate (GL)-containing polyhydroxyalkanoate (PHA) was synthesized in *Escherichia coli* expressing the engineered chimeric PHA synthase PhaC_*AR*_ and coenzyme A transferase. The cells produced poly[GL-*co*-3-hydroxybutyrate (3HB)] with the supplementation of GL and 3HB, thus demonstrating that PhaC_*AR*_ is the first known class I PHA synthase that is capable of incorporating GL units. The triad sequence analysis using ^1^H nuclear magnetic resonance indicated that the obtained polymer was composed of two distinct regions, a P(GL-*ran*-3HB) random segment and P(3HB) homopolymer segment. The random segment was estimated to contain a 71 mol% GL molar ratio, which was much greater than the value (15 mol%) previously achieved by using PhaC1_*P*__*s*_STQK. Differential scanning calorimetry analysis of the polymer films supported the presence of random copolymer and homopolymer phases. The solvent fractionation of the polymer indicated the presence of a covalent linkage between these segments. Therefore, it was concluded that PhaC_*AR*_ synthesized a novel random-homo block copolymer, P(GL-*ran*-3HB)-*b*-P(3HB).

## Introduction

Polyhydroxyalkanoates (PHAs) are bacterial storage polyesters, which are currently used as a biobased replacement for some petroleum-derived plastics (Surendran et al., 2020; Zheng et al., 2020). PHAs have attracted considerable interest in recent years for their potential as a biodegradable material (Morohoshi et al., 2018, 2020), since the polymers degrade well in the environment by the action of PHA depolymerases (Juengert et al., 2018). PHAs possess diverse structures due to their variety in monomer constituents and their copolymerization. The primary structure of PHAs critically influences the physical properties of the material. For example, a random copolymerization of 3-hydroxybutyrate (3HB) and 3-hydroxyhexanoate (3HHx) units with controlled composition is effective for regulating the crystallinity of the polymer so that the P(3HB-*co*-3HHx) copolymer possesses a flexible mechanical property that can be adjusted for various uses, such as a mono-material (Wong et al., 2012), composite (Rebia et al., 2019), or blend (Ivorra-Martinez et al., 2020). Therefore, engineering the PHA structure is important for expanding its range of applications.

The glycolate (GL) unit is an unusual component of PHA, because it does not exist in naturally occurring PHAs (Matsumoto et al., 2011). As a typical example, P(GL-*co*-3HB) is a semitransparent material with pliable property (Matsumoto et al., 2017). In addition, non-enzymatic hydrolytic degradability is an important feature of P(GL-*co*-3HB). Chemically synthesized polyglycolide acid and poly(lactide-*co*-glycolide) are known as highly hydrolytic degradable materials, and they are used in biomedical fields as bioabsorbable materials (Shawe et al., 2006; Pervaiz et al., 2019). Although the bioabsorption of natural PHAs is very slow, natural PHAs have also been studied extensively for their applications in tissue engineering because of their biocompatibility (Basnett et al., 2018; Chen and Zhang, 2018). Artificial PHA P(GL-*co*-3HB) exhibits an intermediate hydrolytic degradability between P(3HB) and poly(lactide-*co*-glycolide), which potentially manipulates the bioabsorption rate of PHAs (Matsumoto et al., 2017).

PHA synthases are the key enzymes that determine the monomeric unit composition of a polymer (Tan et al., 2020). The engineered PHA synthase PhaC1_*P*__*s*_STQK, which belongs to class II PHA synthases and has two point-mutations, possesses extremely broad substrate specificity and plays a central role in the biosynthesis of lactate-based and other 2-hydroxyalkanoate-based PHAs (Taguchi et al., 2008; Taguchi and Matsumoto, 2020). The finding of the glycolyl (GL)-CoA-polymerizing activity of PhaC1_*P*__*s*_STQK was the first discovery of the biosynthesis of a GL-based PHA (Matsumoto et al., 2011). Nevertheless, only PhaC1_*P*__*s*_STQK and a homologous enzyme with the same point mutations are known to produce GL-based PHAs to date. Therefore, new GL-CoA-polymerizing PHA synthase(s) are desired to expand the variety of GL-based PHAs.

The present study aims to examine the GL-incorporating capacity of an engineered PHA synthase, PhaC_*AR*_. PhaC_*AR*_ is a chimeric PHA synthase composed of the N-terminal region of PhaC_*Ac*_ derived from *Aeromonas caviae* and the C-terminal region of PhaC_*Re*_ derived from *Ralstonia eutropha* (*Cupriavidus necator*) (Matsumoto et al., 2009). The junction site of the chimeric enzymes is chosen in their highly conserved and putative random coil regions of each protein and they are fused without inserting a linker region. PhaC_*AR*_ is the first known class I PHA synthase that can efficiently incorporate 2-hydroxybutyrate (2HB) units (Matsumoto et al., 2018a), whereas PhaC_*Re*_ exhibits very little activity toward 2HB-CoA (Han et al., 2011). Moreover, PhaC_*AR*_ possesses a unique function for synthesizing block copolymers (Matsumoto et al., 2018a). *Escherichia coli* expressing PhaC_*AR*_ and propionyl-CoA transferase (PCT) spontaneously produced P(2HB-*b*-3HB) from the mixture of 2HB and 3HB precursors supplemented in the medium. P(2HB-*b*-3HB) is the first structure-proven block PHA. Therefore, the monomer sequence of the obtained polymer is of interest in the attempt to incorporate GL units using PhaC_*AR*_. Indeed, we found PhaC_*AR*_ is the first known class I GL-CoA-polymerizing PHA synthase and that the obtained polymer possesses a unique block sequence consisting of a random segment and a homopolymer segment.

## Materials and Methods

### Plasmids and Culture Conditions

pBSP_*Re*_phaC_*AR*_pct harboring the *phaC*_*AR*_ and PCT genes from *Megasphaera elsdenii* under the control of the *R. eutropha phb* operon promoter and pBSP_*Re*_phaC1_*P*__*s*_STQKpct harboring the *phaC1_*P*__*s*_STQK* instead of the *phaC*_*AR*_ were constructed in a previous study (Matsumoto et al., 2018a). pBSP_*Re*_phaC_*AR*_pctAB, which is a pBSP_*Re*_phaC_*AR*_pct derivative harboring the *phaA* and *phaB* genes from *R. eutropha*, was constructed by inserting the *Sac*I fragment of pGEM’-phbCAB (Matsusaki et al., 2000), which contains the *phaAB* genes, into the *Sac*I site of pBSP_*Re*_phaC_*AR*_pct (Supplementary Scheme S1). *E. coli* JM109 strains harboring these plasmids were cultivated on a 100 mL Luria–Bertani (LB) medium containing 2 wt% glucose, 100 mg/L ampicillin, 5 g/L sodium (*R,S*)-3HB and varied concentrations of sodium GL in a 500 mL shake flask at 30°C for 24 h with reciprocal shaking at 120 rpm.

### Capillary Electrophoresis

The concentration of monomer precursors in the medium was measured using Agilent 7100 CE, a capillary electrophoresis (CE) system, equipped with a capillary tube (HPCE standard cap 50 μm id, 72 cm). The sample was injected for 4 s under a pressure of 50 mbar. The electrophoresis was performed at 25°C and -15 kV, with α-AFQ108 used as the running buffer (Otsuka Electronics Co., Ltd., Osaka, Japan). The sample was detected by a diode array at 400 nm.

### Gas Chromatography

The lyophilized cells (approximately 10 mg) were treated in a solution of 0.5 mL chloroform and 0.5 mL 15% sulfuric acid in ethanol at 100°C for 2 h. The obtained ethyl esters were analyzed by gas chromatography (GC) as described previously (Taguchi et al., 2008).

### Analytical Methods of Polymers

The polymer was extracted from lyophilized cells with chloroform at 60°C for 48 h in a test tube with a screw cap. The cell debris was removed through a 0.2-μm-pore-size polytetrafluoroethylene (PTFE) membrane filter. The chloroform extract was concentrated under air flow in a fume hood. The excess amount (approximately 10 times) of methanol was added to the extract at room temperature to precipitate the polymer. This purified polymer was applied in further analyses.

A differential scanning calorimetry (DSC) sample was prepared on an aluminum pan. A polymer chloroform solution containing approximately 3 mg polymer was applied to the pan and air dried for 2 h, then subsequently *in vacuo* for 24 h at room temperature. The DSC data were recorded on a DSC3+ differential scanning calorimeter (Mettler Toledo). The samples were heated from -50 to 210°C at 20°C/min (the first heating scan). After rapid quenching at -50°C and isothermal incubation for 5 min, the samples were heated to 210°C at 20°C/min for a second heating scan.

Nuclear magnetic resonance (NMR) analysis of the polymers was performed using approximately 5 mg/mL of a polymer solution in CDCl_3_ containing tetramethylsilane, which was dissolved at 60°C and passed through a 0.2-μm-pore-size PTFE membrane filter. The NMR data were recorded on JEOL ECX-400 and ECS-400 spectrometers (JEOL, Japan). The same solution was analyzed using size-exclusion chromatography equipped with two tandem high-performance liquid chromatography columns of Shodex GPC K-806L (Shodex, Japan). The flow rate was 0.8 mL/min; the column oven was kept at 40°C; and the sample volume was 100 μL.

### Solvent Fractionation

The purified polymer samples were fractionated into two fractions via solubility to distinguish a polymer blend and block copolymer (Matsumoto et al., 2018a). The 10 mg polymer samples were dissolved in 4 mL chloroform at 60°C for 15 min. After the solution was cooled to room temperature, 0.5 mL methanol was added to the solution and incubated at room temperature for 1 h. This step was repeated until precipitation was visually observed. As the results, total 5.0 and 4.5 mL methanol was added to the P(GL-*co*-3HB) and P(3HB) solutions, respectively. Then, the suspension was filtrated through a 0.1-μm-pore-size PTFE membrane filter. The precipitated fraction refers to the polymer on the filter that was recovered in chloroform, whereas the soluble fraction was the polymer solution that dried up after passing through the filter.

## Results

### Production of the GL-Containing Polymer in *E. coli* Harboring the *phaC*_*AR*_ Gene

The attempts to synthesize P(GL-*co*-3HB) in *E. coli* JM109 tested the GL-incorporating capacity of PhaC_*AR*_. Two 3HB-CoA supplying pathways were used: the CoA transferring pathway catalyzed by PCT from the 3HB supplemented in the medium (pBSP_*Re*_phaC_*AR*_pct) and the well-known dimerization pathway of acetyl-CoA catalyzed by β-ketothiolase (PhaA) and acetoacetyl-CoA reductase (PhaB) (pBSP_*Re*_phaC_*AR*_pctAB). First, the cells harboring the pBSP_*Re*_phaC_*AR*_pct were grown on the GL and 3HB. The *phaC1_*P*__*s*_STQK* gene served as a control. As a result, a polymer containing GL units was obtained in both constructs (Table 1), which indicates that PhaC_*AR*_ possesses GL-CoA-polymerizing activity. In addition, the GL molar ratio in the polymer synthesized by PhaC_*AR*_ was higher than that of PhaC1_*P*__*s*_STQK. Therefore, PhaC_*AR*_ has greater GL-incorporating capacity than that of PhaC1_*P*__*s*_STQK. The slightly decreasing trend of the cell dry weight (CDW) with an increase in the GL concentration was due to the toxicity of the GL in the medium and a decrease in polymer production. A GL concentration of 4 g/L was used in further study, because a greater increase in the GL concentration (12 g/L) exhibited no significant effect on the GL molar ratio. *E. coli* did not grow at 20 g/L GL concentration (data not shown).

**TABLE 1:** Glycolate-based polymer synthesis in *E. coli* JM109 expressing different PHA synthases, PhaC1_*P*__*s*_STQK and PhaC_*AR*_^*a*^.

**PHA synthase**	**GL-Na (g/L)**	**CDW (g/L)**	**Polymer production (g/L)**	**Monomer composition (mol%)**	
				**GL**	**3HB**
PhaC1_*P*__*s*_STQK	0	3.7 ± 0.14	0.41 ± 0.01	0 ± 0	100 ± 0
	4	3.3 ± 0.04	0.31 ± 0.07	17 ± 1.4	83 ± 1.4
	12	2.8 ± 0.06	0.36 ± 0.01	16 ± 0.7	84 ± 0.7
PhaC_*AR*_	0	3.8 ± 0.07	0.56 ± 0.08	0 ± 0	100 ± 0
	4	3.6 ± 0.08	0.42 ± 0.12	20 ± 3.0	80 ± 3.0
	12	3.4 ± 0.12	0.45 ± 0.01	23 ± 0.5	77 ± 0.5

*^*a*^The data are the average ± standard deviations of three independent trials.*

The cells harboring pBSP_*Re*_phaC_*AR*_pctAB produced P(8 mol% GL-*co*-3HB) from the glucose and GL (Supplementary Table S1), which indicates that the supplementation of 3HB in the medium can be replaced by PhaAB. In further investigations of the present study, however, 3HB supplementation was used because of its higher GL molar ratio in the polymer, which facilitated the structural analysis of the polymers.

### The Time-Course of GL-Based Polymer Production Using PhaC_*AR*_

**FIGURE 1:**
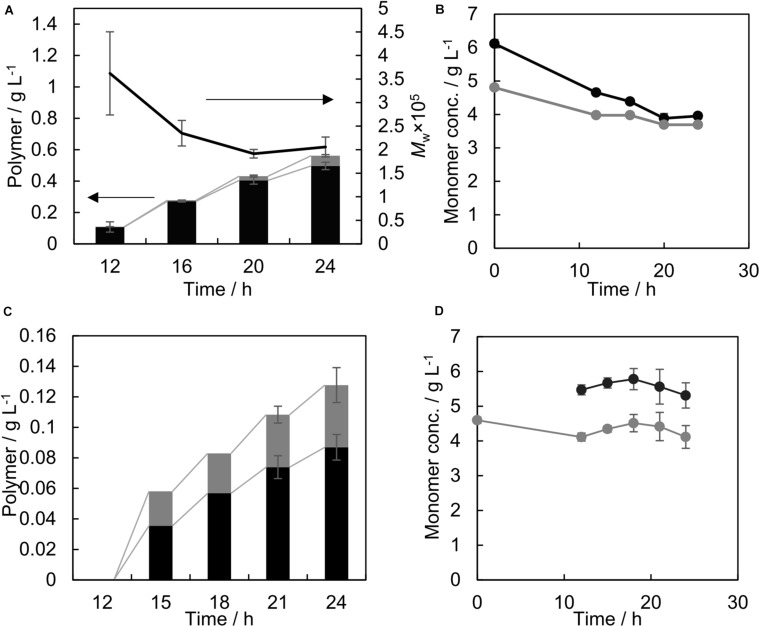
Time course of GL-based polymer production by PhaC_*AR*_ **(A,C)** and the concentration of monomer precursors in the medium **(B,D)**, with the precursor added at different times. Black and gray bars indicate 3HB and GL units in the polymer, respectively, and the line graph indicates the weight-average molecular weight of the polymer **(A)**. Black and gray circles indicate the concentrations of 3HB and GL in the medium, respectively **(B,D)**. Both precursors 3HB and GL were added at 0 h **(A,B)**; GL was added at 0 h and 3HB was added at 12 h **(C,D)**. A 12 h sample was taken immediately after the 3HB addition.

The time-course of GL-based polymer synthesis using PhaC_*AR*_ was monitored. The GL molar ratio was relatively low at 12 h and exhibited an increasing trend corresponding with the cultivation time (Figure 1A). This indicates that a P(3HB)-like polymer was synthesized during the first 12 h. The concentrations of the precursors in the medium did not significantly decrease during cultivation (Figure 1B). Thus, the change in the monomer composition was not due to the precursor concentrations in the medium. PhaC1_*P*__*s*_STQK exhibited a similar trend during the time-course of polymer production (Supplementary Figure S1).

To eliminate the time-dependent change of the monomer composition, 3HB was added at 12 h (Figure 1C). Under these conditions, no polymer was produced during the initial 12 h. Consequently, the monomer composition stayed nearly constant throughout the cultivation process (Figure 1C), 41 mol% GL at 18–24 h). The GL molar ratio in the polymer considerably increased compared to that under the conditions shown in Table 1. The precursor consumptions in the medium were almost negligible (Figure 1D). Thus, the precursor concentrations did not influence the monomer composition. The obtained polymer was used in further studies.

Under the condition of Figure 1C, no polymer was detected at 12 h in the chloroform extract, which could be due to the insolubility of the polyglycolate homopolymer in chloroform. Therefore, the whole cell at 12 h was subjected to GC analysis. As a result, 0.05 ± 0.01 g/L GL was detected in the 2.5 ± 0.0 g/L CDW, which suggests that a small amount of polyglycolate was synthesized, although the product was not contained in the extracted polymer samples. Further analysis is needed to characterize the product.

### Differences in the ^1^H NMR of the Copolymer Synthesized by PhaC_*AR*_ and PhaC1_*P*__*s*_STQK

**FIGURE 2:**
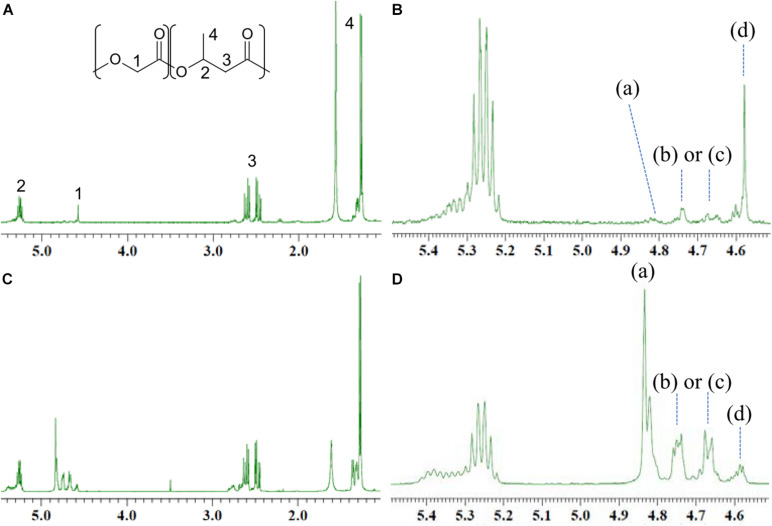
^1^H NMR analysis of the GL-based copolymers containing 11 mol% GL synthesized by PhaC1_*P*__*s*_STQK **(A,B)** and 40 mol% GL synthesized by PhaC_*AR*_ **(C,D)**.

GL-based polymers synthesized by PhaC_*AR*_ and PhaC1_*P*__*s*_STQK were analyzed by ^1^H NMR. The resonance at 5.2–5.4 ppm was ascribed to the methine proton of 3HB units. The low-field shift of the resonance was due to the presence of a GL-3HB^∗^ dyad in the polymer. The methylene proton of the GL units exhibited four characteristic resonances at 4.5–4.9 ppm. Based on the analogy to poly(lacatate-*co*-3HB) (Yamada et al., 2009), these four resonances were ascribed to GL-GL^∗^-GL (a), GL-GL^∗^-3HB or 3HB-GL^∗^-GL [(b) or (c)], and 3HB-GL^∗^-3HB (d), respectively (Matsumoto et al., 2017). Notably, the intensity of resonance (d) was highest among the signals in the polymer synthesized by PhaC1_*P*__*s*_STQK (Figure 2B), which indicates that the 3HB-GL-3HB triad is abundant in the copolymer. In contrast, resonance (a) was strongest in the polymer synthesized by PhaC_*AR*_ (Figure 2D), which indicates the abundance of the GL-GL-GL triad in the copolymer. A similar resonance pattern was observed when 3HB was supplied at 0 h (Supplementary Figure S2B under the conditions shown in Figure 1A). These results indicate that the copolymers synthesized by PhaC_*AR*_ and PhaC1_*P*__*s*_STQK differed in terms of the monomer sequence in the polymer chain. Furthermore, the copolymer synthesized by PhaC_*AR*_ contains an irregularly heterogenous structure, which was revealed by the detailed analysis of the results as discussed below.

### Sequence Heterogeneity Analysis of the Copolymers

The ^1^H NMR pattern of the GL units can be used to determine the heterogeneity of the monomer sequence in a polymer chain. For example, the strong intensity of the GL-GL-GL triad indicates the presence of a GL-rich region in the polymer chain. The local GL molar ratio in the GL-rich region can be quantitatively estimated based on the relative intensity of the four triad signals (Table 2).

**TABLE 2:** Monomer sequence analysis of P(GL-*co*-3HB)s synthesized by PhaC_*AR*_ and PhaC1_*P*__*s*_STQK based on the ^1^H NMR intensity.

**No.**	**Polymer**	**GL ratio (mol%)**	**3HB ratio (mol%)**	**Relative ^1^H NMR intensity of GL units in the triad sequences**			
				***a***	***b***	***c***	***d***
1	P(GL-*co*-3HB) synthesized by PhaC_*AR*_ (determined)	40 ± 4	60 ± 4	0.51 ± 0.02	0.21 ± 0.01	0.21 ± 0.01	0.08 ± 0.009
2	Ideal random copolymer (calculated)	71	29	0.50	0.21	0.21	0.08
3	P(GL-*co*-3HB) synthesized by PhaC1_*P*__*s*_STQK (determined)	11 ± 1	89 ± 1	0.07 ± 0.10	0.15 ± 0.01	0.12 ± 0.00	0.66 ± 0.014
4	Ideal random copolymer (calculated)	18	82	0.03	0.15	0.15	0.67

*The determined data are the average ± standard deviations of three independent trials.*

The value ***a*** is defined as the relative intensity of the GL-GL-GL triad over the total peak intensity ascribed to the GL units (Figure 2). The function *Area* is defined as the ^1^H NMR peak area of the molecular species. The values ***b***, ***c***, and ***d*** are similarly defined based on the peak intensities of (b), (c), and (d) (Table 2).

$$\text{a}=\frac{A\,r\,e\,a\,\left[\left(a\right)\right]}{A\,r\,e\,a\,\left[\left(a\right)+\left(b\right)+\left(c\right)+\left(d\right)\right]}\left(0\leq a\leq 1\right)$$

Given an ideal random copolymer with a GL rate of ***x*** (mol/mol) over the total polymer, the abundance of the four triad sequences ***a***–***d*** is calculated by the following equations. Here, 1-***x*** indicates the 3HB rate (mol/mol) over the total polymer.

(1) $$\text{a}=x^{2}$$

(2) b⁢a⁢n⁢d⁢c=x⁢(1-x)

(3) $$\text{d}={\left(1-x\right)}^{2}$$

Based on the ^1^H NMR of P(40 mol% GL-*co*-3HB) synthesized by PhaC_*AR*_, the ***a***∼***d*** values were experimentally determined (Table 2, No. 1). Surprisingly, these values were nearly identical to those of an ideal random copolymer with 71 mol% GL (Table 2, No. 2, calculated using formulas (1)–(3) when ***x*** = 0.71). This means that the P(40 mol% GL-*co*-3HB) contains a considerably rich GL region, which is a nearly ideal random copolymer with a local GL molar ratio of 71 mol%. Thus, the GL-rich region is referred to as the P(GL-*ran*-3HB) segment. Consequently, it was rationally presumed that the copolymer contains a region(s) composed of mostly 3HB units, which is referred to as the P(3HB) segment. The molar ratio of the P(3HB) segment over the total polymer was calculated by subtracting the amount of 3HB units in the P(GL-*ran*-3HB) segment from the total 3HB units as shown in the following equation. Here, function [*n*] indicates the molar ratio of *n*. ***x*** is the local GL rate (mol/mol) in the P(GL-*ran*-3HB) segment; and 1-***x*** is the local 3HB rate (mol/mol) in the P(GL-*ran*-3HB) segment.

$$\left[t\,o\,t\,a\,l\,3\,H\,B\right]-\left[t\,o\,t\,a\,l\,G\,L\right]\times \frac{1-x}{x}=\left[P\,\left(3\,H\,B\right)\,s\,e\,g\,m\,e\,n\,t\right]$$

**FIGURE 3:**
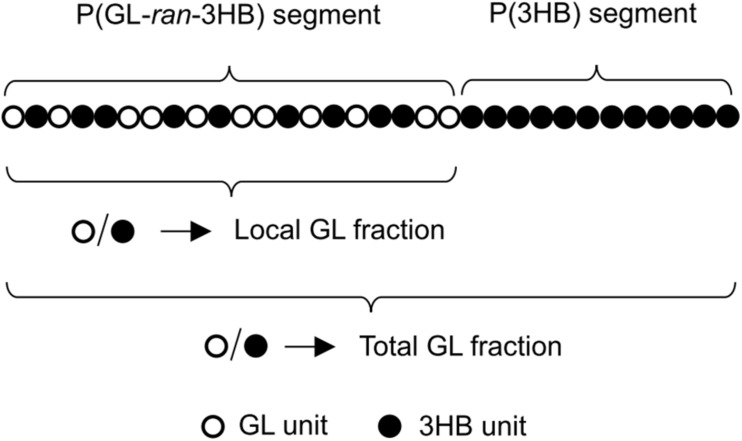
The proposed random-homo block monomer sequence of P(GL-*co*-3HB) synthesized by PhaC_*AR*_.

Using the values of 0.71 for ***x***, 0.6 for [total 3HB], and 0.4 for [total GL], the [P(3HB) segment] was estimated to be 0.44 (mol/mol). The high intensity of the resonance occurring at 5.2 ppm, which is ascribed to the methine proton of the 3HB units in the 3HB-3HB^∗^ dyad, is consistent with the interpretation. Overall, it was concluded that the copolymer synthesized by PhaC_*AR*_ is composed of two segments, P(71 mol% GL-*ran*-3HB) and P(3HB), and the ratio of these segments is 56:44 (mol/mol) (Figure 3).

In contrast to PhaC_*AR*_, PhaC1_*P*__*s*_STQK synthesized the copolymer with a large ***d***, which indicates an abundant 3HB-GL-3HB triad in the polymer chain (Table 2, No. 3). The ***a–d*** values were close, but not equal, to the values that were calculated from an ideal random copolymer with 18 mol% GL (***x*** = 0.18) (No. 4). The calculated value (18 mol%) was slightly greater than the actual GL molar ratio (11 mol%). These results indicate that the copolymer synthesized by PhaC1_*P*__*s*_STQK is a nearly random copolymer, but the monomer sequence is not ideally random and contains a slightly GL-rich region.

### Thermal Property Analysis of P(GL-*co*-3HB)s Synthesized by PhaC1_*P*__*s*_STQK and PhaC_*AR*_

**FIGURE 4:**
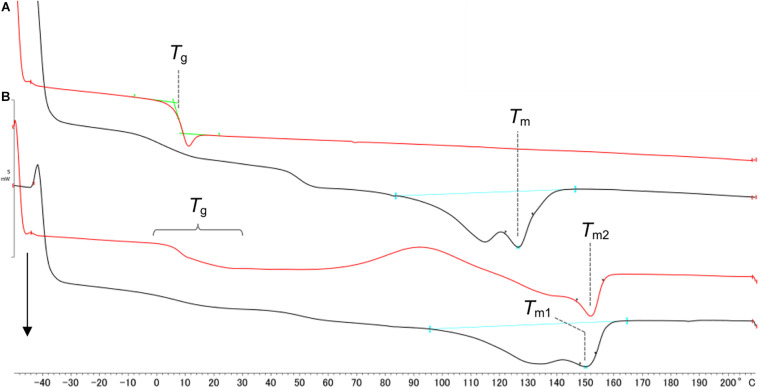
DSC analysis of P(GL-*co*-3HB)s synthesized by PhaC1_*P*__*s*_STQK **(A)** and PhaC_*AR*_ **(B)**. The black line indicates the 1^st^ heating scan. The red line indicates the 2^nd^ heating scan. The arrow indicates endothermic reactions. **(A)** *T*_*m*_ = 126.3°C, Δ*H*_*m*_ = 23.0 J/g and *T*_*g*_ = 7.6°C. **(B)** *T*_*m1*_ = 151.4°C, Δ*H*_*m*_ = 34.6 J/g (1^st^ heating scan), and *T*_*m2*_ = 151.6°C (2^nd^ heating scan). 3HB was supplemented at 12 h to produce a polymer by PhaC_*AR*_.

The ^1^H NMR analysis indicated that P(GL-*co*-3HB)s synthesized by PhaC1_*P*__*s*_STQK and PhaC_*AR*_ has distinct structures in terms of the monomer sequence. The interpretation was verified by the thermal properties of the polymers. The whole polymer without solvent fractionation was used for the analysis. P(11 mol% GL-*co*-3HB) synthesized by using PhaC1_*P*__*s*_STQK exhibited a small melting peak at 126°C (Figure 4A), which was lower than that of P(3HB) (176°C) (Yamada et al., 2009), and no melting peak was detected in the second heating scan. The low and slow crystallization agreed with the randomly copolymerized structure indicated by the ^1^H NMR analysis. The copolymer exhibited a glass transition temperature (*T*_*g*_) at 7.6°C, which is consistent with the previous result (Matsumoto et al., 2017).

**FIGURE 5:**
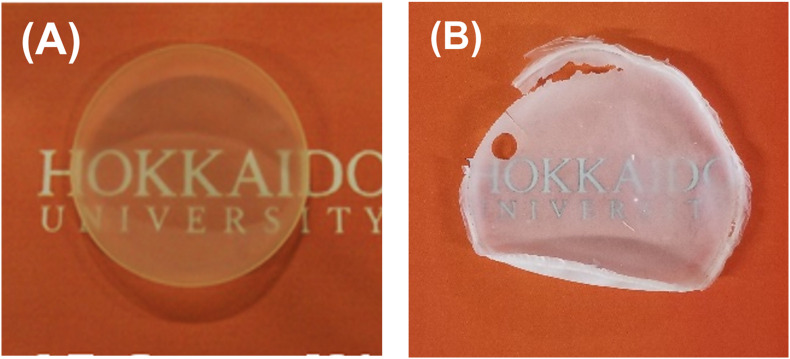
Solvent-cast films of P(GL-*co*-3HB)s. P(16 mol% GL-*co*-3HB) synthesized by PhaC1_*P*__*s*_STQK **(A)**. P(14 mol% GL-*co*-3HB) synthesized by PhaC_*AR*_ **(B)**.

In contrast, the copolymer synthesized by using PhaC_*AR*_ exhibited a larger melting peak at 151.4°C, and a melting peak was also detected in the 2^nd^ heating scan (Figure 4B). The high and fast crystallization of the polymer should be due to the presence of a P(3HB) segment in the polymer. The degree of crystallinity of the polymer synthesized by PhaC_*AR*_ (24%), which was estimated using the enthalpy of fusion of 100% P(3HB) crystal (146 J/g) (Barham et al., 1984), was 1.5-fold greater than the crystallinity of the polymer synthesized by PhaC1_*P*__*s*_STQK (16%). The copolymer synthesized by PhaC_*AR*_ exhibited a complicated *T*_*g*_ shift (Figure 4B), which indicates that the polymer possesses multiple *T*_*g*_s. These results show a good agreement with the model in Figure 3. In fact, the crystalline property of the polymer synthesized by PhaC_*AR*_ was observed as a film opacity, which was in contrast with the semitransparent P(GL-*co*-3HB) film synthesized by PhaC1_*P*__*s*_STQK with similar GL molar ratio (Figure 5), and a low extensio to break (7%, Supplementary Figure S3).

### Solvent Fractionation

The copolymer synthesized by using PhaC_*AR*_ contained two distinguishable segments. To determine whether these segments could be a block copolymer or polymer blend, the copolymer was subjected to solvent fractionation. Because no sufficient difference in the solubility was found between P(GL-*ran*-3HB) and P(3HB), the polymer solution in chloroform was partially precipitated by adding methanol. This fractionation method is based on the principle that the solubility of polymers in organic solvents is dependent on their molecular weight. In fact, the insoluble fraction of the partially precipitated P(3HB) possesses a higher molecular weight than that of the soluble (non-precipitated) fraction (Table 3).

**TABLE 3:** Solvent fractionation of P(GL-*co*-3HB) synthesized by PhaC_*AR*_.

**Sample**		**Recovered amount (mg)**	***M*_*w*_ (×10^5^)**	***M*_*w*_/*M*_*n*_**	**Total GL ratio (mol%)**	**Local GL ratio (mol%)**	**P(3HB) segment (mol%)**
P(3HB)	Before fractionation		1.8	2.3			
	Precipitated fraction	2	2.0	1.8			
	Soluble fraction	9	1.7	2.0			
P(GL-*co*-3HB) synthesized by PhaC_*AR*_	Before fractionation		1.9	4.6	30	68	55
	Precipitated fraction	2	0.8	2.1	53	75	30
	Soluble fraction	8	2.1	5.0	24	64	63

*Ten milligrams of polymers were used for each condition. The GL molar ratio and P(3HB) segment were determined by ^1^H NMR analysis (Supplementary Figure S3).*

After the addition of methanol, P(GL-*co*-3HB) synthesized by PhaC_*AR*_ was separated into two fractions by solvent fractionation: precipitated and soluble fractions. The precipitated fraction showed higher total and local GL molar ratios, which was determined based on the ^1^H NMR (Supplementary Figure S4) using the same method as shown in Table 2, than those of the soluble fraction (Table 3), which indicates that GL units promoted the precipitation of the polymer. This result agrees with the fact that polyglycolic acid is not soluble in chloroform and in methanol. The precipitated and soluble fractions both contained the P(3HB) segment. Notably, the molecular weight of the precipitated fraction (*M*_*w*_ = 80,000) was lower than that of the soluble fraction (*M*_*w*_ = 210,000). The postulation that the sample is a blend of P(GL-*ran*-3HB) and P(3HB) leads to a contradicting interpretation that P(3HB) with relatively low *M*_*w*_ was readily precipitated. Therefore, it is likely that the P(3HB) segment in the precipitated fraction is covalently linked to the P(GL-*ran*-3HB) segment.

Considering the results of the solvent fractionation and thermal property analysis together, it was concluded that the copolymer synthesized by PhaC_*AR*_ is a random-homo block copolymer, P(GL-*ran*-3HB)-*b*-P(3HB) (Figure 3). Currently, the number of segments per polymer chain, namely diblock, triblock, etc., is unknown.

## Discussion

The present study demonstrates that PhaC_*AR*_ is the first known GL-incorporating class I PHA synthase. It was concluded that PhaC_*AR*_ possesses a higher GL-incorporating capacity than that of PhaC1_*P*__*s*_STQK, because PhaC_*AR*_ synthesized the P(GL-*ran*-3HB) segment, which was estimated to contain 71 mol% GL, whereas PhaC1_*P*__*s*_STQK can incorporate a much lower GL molar ratio (Table 2). The contrasting results of two PHA synthases suggest that the high local GL ratio is due to the enzymatic properties of PhaC_*AR*_ because the promoter, monomer supplying enzyme and culture conditions are the same. The incorporation of GL units by PhaC_*AR*_ had no considerable effect on the molecular weight of the polymer (Table 3) in contrast to the case of PhaC1_*P*__*s*_STQK, in which the molecular weight decreases as GL ratio increases (Matsumoto et al., 2017).

Moreover, the obtained polymer was a unique block PHA, P(GL-*ran*-3HB)-*b*-P(3HB). PhaC_*AR*_ was previously shown to synthesize P(2HB-*b*-3HB), which is composed of homopolymer segments. These results raise a question about why a random segment was generated by the same set of enzymes PhaC_*AR*_ and PCT. One possible factor for the generation of a random segment is the copolymerization kinetics of PHA synthase. It was previously demonstrated that the *in vitro* activity of PhaC1_*P*__*s*_STQK toward lactyl-CoA was lower than that toward 3HB-CoA under single substrate conditions. However, when two substrates (each 0.2 mM) were combined, lactyl-CoA was consumed faster than 3HB-CoA (Matsumoto et al., 2018b). A similar mechanism could take place in the GL-CoA and 3HB-CoA copolymerization by PhaC_*AR*_. An *in vitro* analysis of PhaC_*AR*_ will be needed in a future study. The *T*_*g*_ of the polymer product also influences polymer synthesis. Our recent study revealed that P(2HB) biosynthesis is efficient when the cultivation temperature is higher than the *T*_*g*_ of the polymer (Matsumoto and Kageyama, 2019). Given the fact that the random copolymer possesses a lower *T*_*g*_ than that of polyglycolate (35–40°C), P(GL-*ran*-3HB) chains are more readily synthesized than the homopolymer. In other words, cultivation temperature could be a parameter for regulating GL ratio in P(GL-*ran*-3HB) segment.

In general, characteristic and useful properties of block copolymers are attributable to the linkage between segments with contrasting properties, such as soft and hard segments (Lendlein et al., 1998). In fact, studies on chemically synthesized block copolymers typically select segments with distinct structures; for example, polyester and polysaccharide segments (Isono et al., 2018). PHAs were also chemically conjugated with PHAs (Dai et al., 2009) and/or other types of polymers, such as poly(ε-caprolactone), to improve the flexibility of the material (Saad et al., 1999). In contrast, wholly biosynthesized PHAs are all composed of hydroxyalkanoate units linked via ester bonds. Therefore, the selection of segments with distinct properties is a challenging requirement in the molecular design of block PHAs. In this light, the technique to synthesize a block PHA containing a random segment is useful to control the physical properties of each segment. However, P(GL-*ran*-3HB)-*b*-P(3HB) was processed into a stiff film as was P(3HB) (Figure 5 and Supplementary Figure S3). The stiff property was presumably due to the high ratio of the P(3HB) segment. Therefore, the regulation of P(3HB) segment ratio is next target to improve the physical property of the material.

The promoter used in this study is derived from *phb* operon of *R. eutropha*, which expresses in *E. coli* without induction under the conditions used in this study. The use of inducible promoter might be useful for optimizing the polymer production. The expression levels of PHA biosynthetic genes reportedly influenced the production and molecular weight of P(3HB) (Hiroe et al., 2012). In our previous study, we utilized the *lac* promoter for production of P(lactate-*co*-3HB) in *E. coli*. Interestingly, the highest polymer production was achieved with relatively low expression conditions (Hori et al., 2020). Although the mechanism behind the phenomenon has not been elucidated, the results suggest that the expression level of PHA biosynthetic enzyme is not a rate-determining step of P(lactate-*co*-3HB) production. Further study will be needed for efficient production of the block PHAs.

## Conclusion

PhaC_*AR*_ was found to be the first known class I GL-CoA-polymerizing PHA synthase. PhaC_*AR*_ synthesized the unique random-homo block PHA consisting of P(GL-*ran*-3HB)-*b*-P(3HB). The finding of the random segment-containing block PHA has the potential to expand the molecular design of polymers with a variety of properties. In addition, the GL-rich structure synthesized by PhaC_*AR*_ is useful as hydrolytically degradable material.

## Data Availability Statement

All datasets generated for this study are included in the article/Supplementary Material, further inquiries can be directed to the corresponding author.

## Author Contributions

SA performed the experiments and wrote the original draft. SS and RM performed the experiments. TO and MZ contributed to the scientific discussion and the manuscript writing. KM conceived and designed the study and wrote the manuscript. All authors revised the manuscript and approved the final manuscript.

## Conflict of Interest

The authors declare that the research was conducted in the absence of any commercial or financial relationships that could be construed as a potential conflict of interest.

## Abbreviations

GL: glycolate
3HB: 3-hydroxybutyrate
LA: lactate
2HB: 2-hydroxybutyrate
PCT: propionyl-CoA transferase.
