# Supplementary material for: Biosynthesis of Random-Homo Block Copolymer Poly[Glycolate-ran-3-Hydroxybutyrate (3HB)]-b-Poly(3HB) Using Sequence-Regulating Chimeric Polyhydroxyalkanoate Synthase in Escherichia coli
Source: Front Bioeng Biotechnol. 2020 Dec 8;8:612991. doi: 10.3389/fbioe.2020.612991 (PMC7752996; doi:10.3389/fbioe.2020.612991)
Supplement: Supplementary file 1 [file Data_Sheet_1.PDF]

Supporting Information

for

Biosynthesis of random-homo block copolymer poly[glycolate-*ran*-3-hydroxybutyrate (3HB)]-*b*-poly(3HB) using sequence-regulating chimeric polyhydroxyalkanoate synthase in *Escherichia coli*

Shuzo Arai[1], Sayaka Sakakibara[1], Robin Mareschal[2,3], Toshihiko Ooi[4], Manfred Zinn[3] and Ken'ichiro Matsumoto[4,*]

[1]Graduate School of Chemical Sciences and Engineering, Hokkaido University, Sapporo, Japan, [2]Department of Engineering, Hokkaido University, Sapporo, Japan, [3]Institute of Life Technologies, University of Applied Sciences and Arts Western Switzerland (HES-SO Valais-Wallis), Sion, Switzerland, and [4]Division of Applied Chemistry, Faculty of Engineering, Hokkaido University, Sapporo, Japan.

Scheme S1

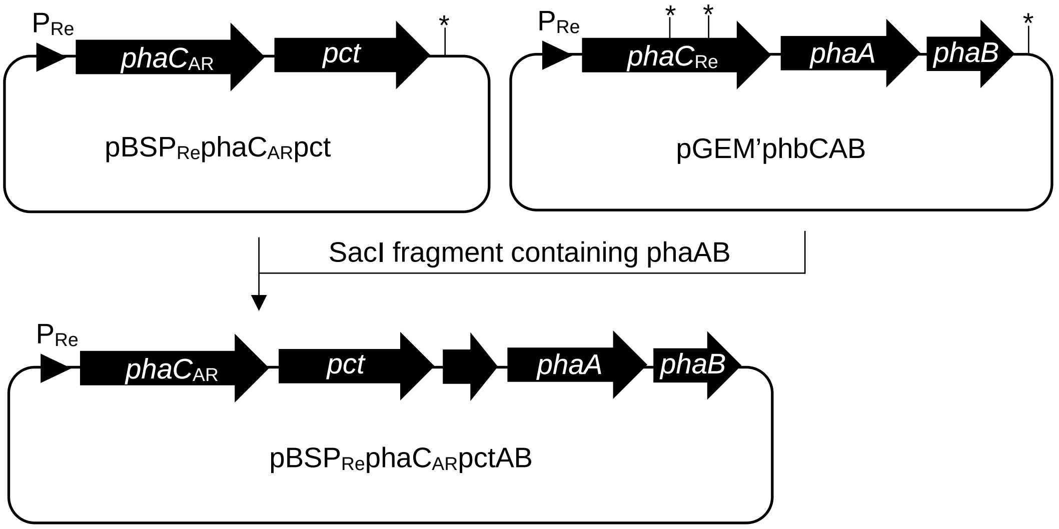

* SacI site

Table S1. P(GL-*co*-3HB) production in *E. coli* harboring pBSP$_{Re}$phaC$_{AR}$pctAB grown on glucose and GL

GL	Cell dry weight (g/L)	Polymer production (g/L)	GL (mol%)	3HB (mol%)
0	5.2	1.5	0	100
5	4.2	0.58	8	92
10	4.7	1.2	7	93

The glucose concentration was 10 g/L.

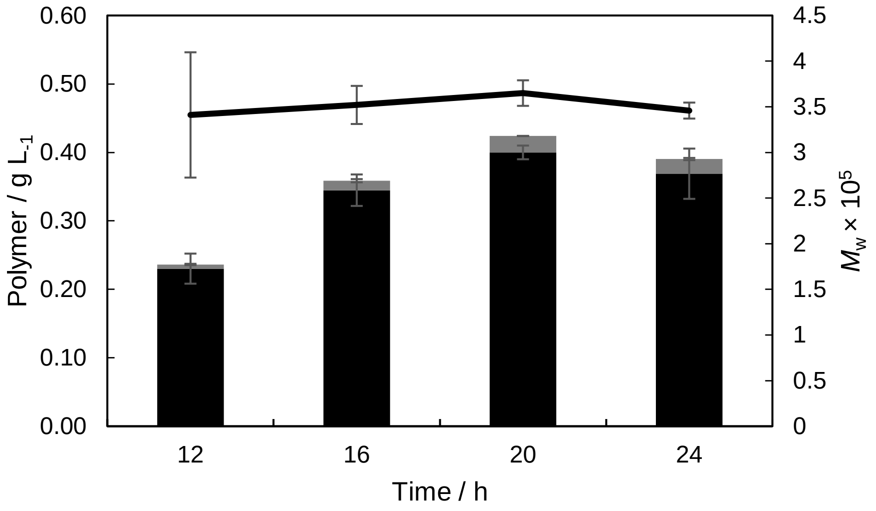

Figure S1. The time-course of P(GL-*co*-3HB) production by PhaC1$_{Ps}$STQK. The polymer was produced with the supplementation of 4 g/L sodium GL. GL and 3HB were supplemented at 0 h. Black bar, 3HB; gray bar, GL. The line graph indicates the weight average molecular weight. The data are the average ± standard deviations of three trials. Doublet data is marked at 20 h.

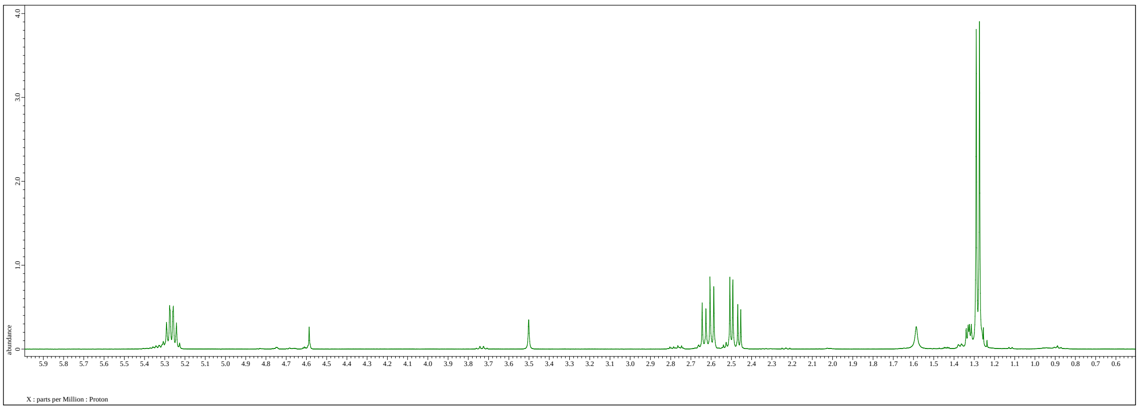

(A): P(GL-*co*-3HB) synthesized by PhaC1$_{Ps}$STQK at 24 h

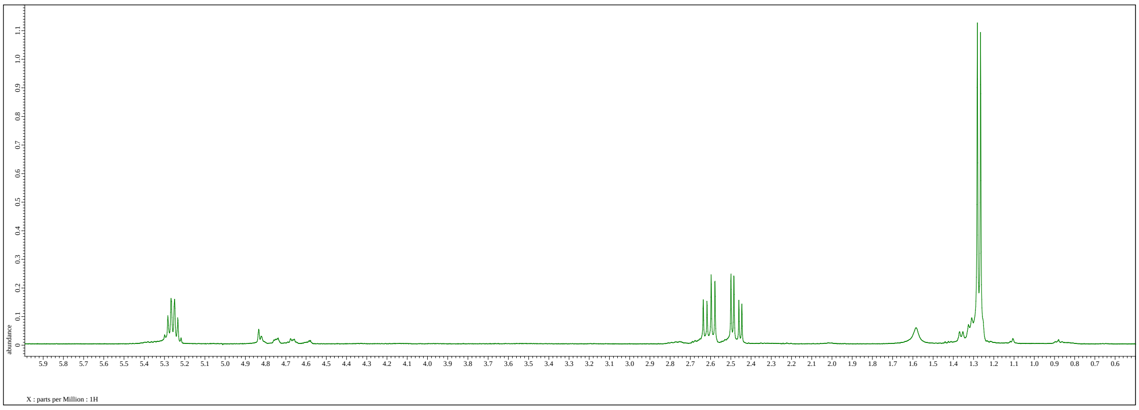

(B): P(GL-*co*-3HB) synthesized by PhaC$_{AR}$ at 24 h produced with the initial addition of GL and 3HB

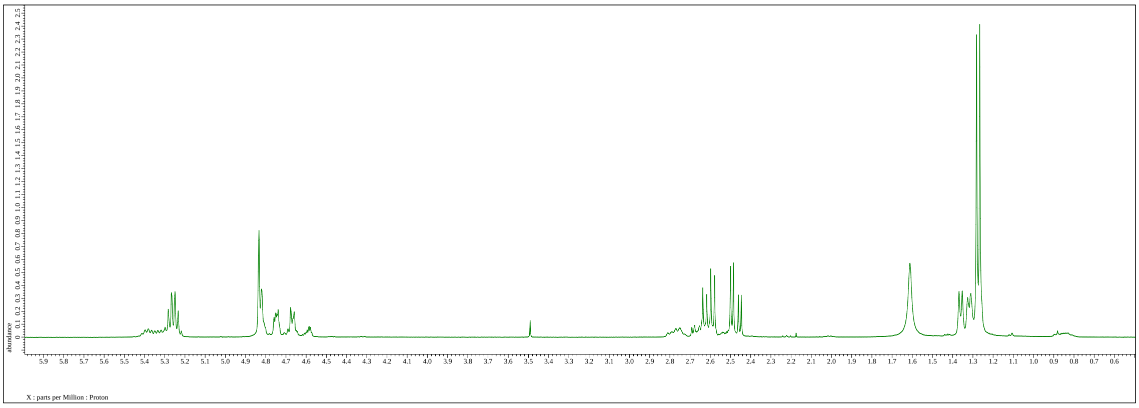

(C): P(GL-*co*-3HB) synthesized by PhaC$_{AR}$ at 24 h produced with the 3HB addition at 12 h

Figure S2. ^{1}H NMR analysis of P(GL-*co*-3HB)s.

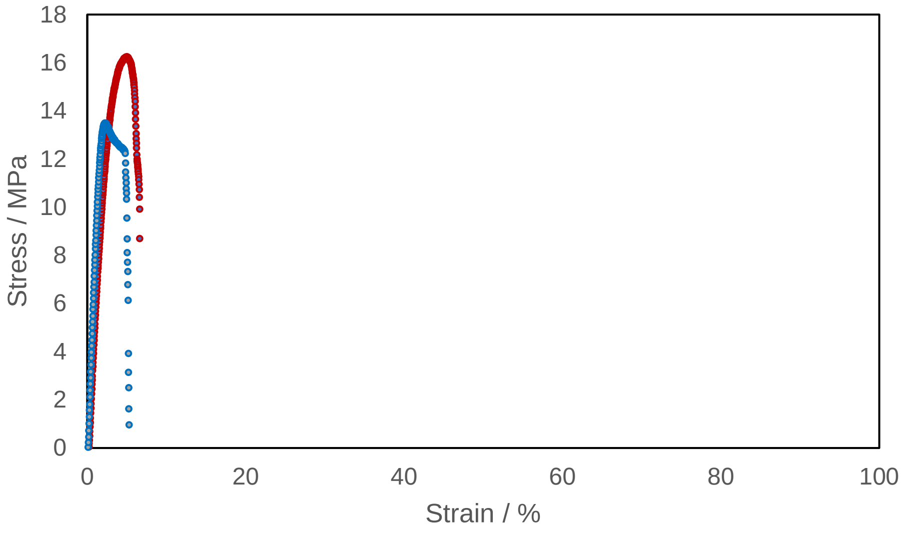

Figure S3. Stress/strain test of solvent-cast films of P(3HB) (blue) and P(GL-*ran*-3HB)-*b*-P(3HB) containing 20 mol% total GL units (red).

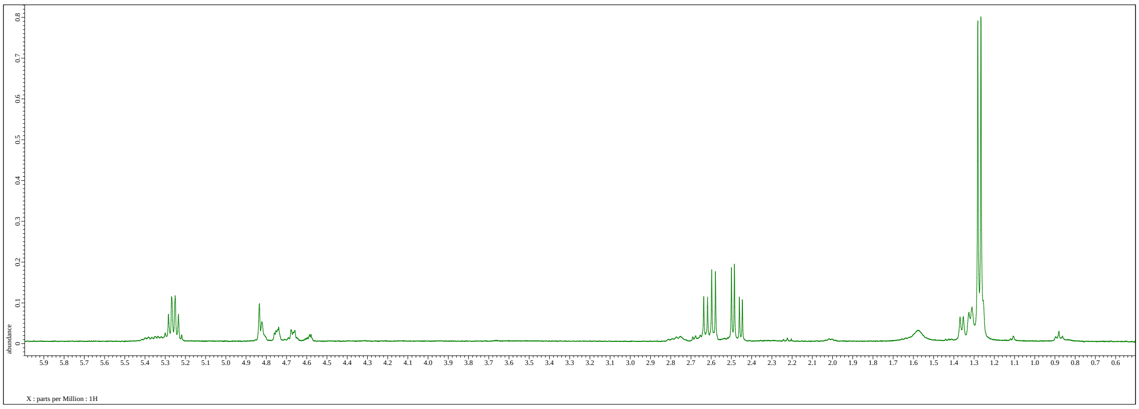

Original P(GL-*co*-3HB) synthesized by $PhaC_{AR}$

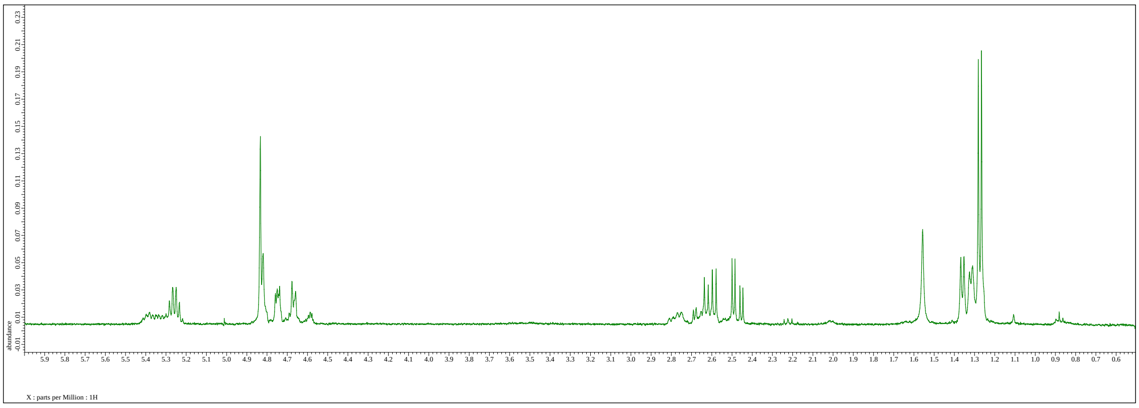

Precipitated fraction of P(GL-*co*-3HB) synthesized by $PhaC_{AR}$

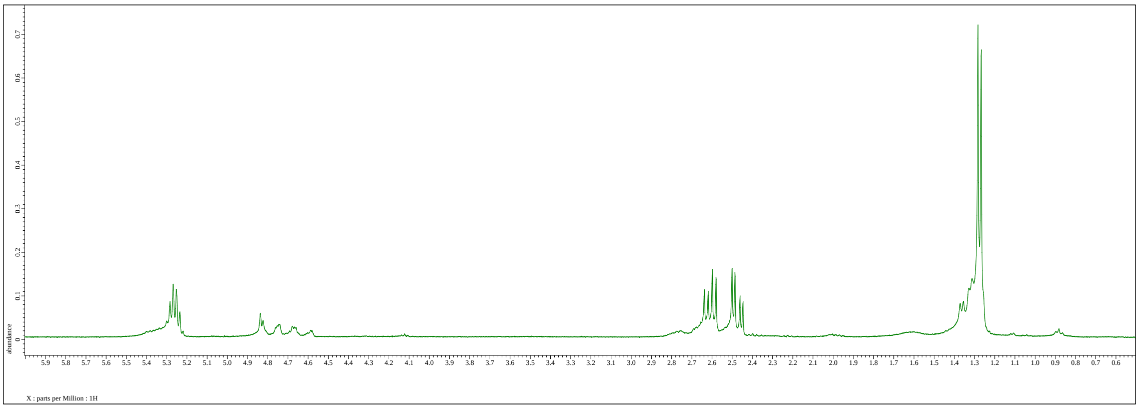

Soluble fraction of P(GL-*co*-3HB) synthesized by $PhaC_{AR}$

Figure S4. ^{1}H NMR analysis of solvent-fractionated P(GL-*co*-3HB) synthesized by $PhaC_{AR}$. The chloroform solution of the original polymer was partially precipitated by adding methanol.
